# Development of Nested Polymerase Chain Reaction with Novel Specific Primers for Detection of *Tritrichomonas muris* Infection in Laboratory Mice

**DOI:** 10.3390/ani13203177

**Published:** 2023-10-11

**Authors:** Hongbo Zhang, Nan Zhang, Jianhua Li, Panpan Zhao, Xin Li, Xiaocen Wang, Xu Zhang, Bao Yuan, Fei Gao, Pengtao Gong, Xichen Zhang

**Affiliations:** 1 Key Laboratory of Zoonosis Research, Ministry of Education, College of Veterinary Medicine, Jilin University, Changchun 130062, China; zhanghb320563@163.com (H.Z.); n_zhang@jlu.edu.cn (N.Z.); jianhuali7207@163.com (J.L.); zpp13796033057@163.com (P.Z.); jlulixin0928@163.com (X.L.); wangxiaocen2016@163.com (X.W.); zhangxu1311@163.com (X.Z.); 2 College of Animal Sciences, Jilin University, Changchun 130062, China; yuan_bao@jlu.edu.cn (B.Y.); gaofei1986@jlu.edu.cn (F.G.)

**Keywords:** *Tritrichomonas muris*, SSU rRNA, primer, nested PCR, laboratory mice

## Abstract

**Simple Summary:**

*Tritrichomonas muris* (*T. muris*) mainly parasitizes the ceca of different rodent species worldwide. To date, there are no ideal methods for the detection of *T. muris* infections in laboratory mice. This study developed a nested PCR reaction using a novel specific primer based on the conserved regions of the SSU rRNA gene of *T. muris* for a molecular epidemiological survey (investigation) of *T. muris* infections in laboratory mice. The results showed that the nested PCR system has higher sensitivity, reliability, and specificity. The nested PCR system showed an infection rate of *T. muris* of 18.96% (58/306), which was higher than the infection rate of 14.05% (43/306) that was detected via smear microscopy in fecal samples from five mouse strains. The sensitivity and specificity of nested PCR in detecting *T. muris* was found to be 100%, and it demonstrated a 26% increase in diagnostic sensitivity compared to the smear microscopy method. The present study provides a new method for the molecular epidemiological investigation of *T. muris* infections in laboratory mice.

**Abstract:**

A variety of rodent ceca are parasitized by *Tritrichomonas muris* (*T. muris*), a flagellated protozoan. To date, there are no ideal methods for the detection of *T. muris* infections in laboratory mice; thus, new molecular methodologies for its specific detection need to be developed. In this study, using staining and SEM, it was observed that *T. muris* has a pear-shaped body and contains three anterior flagella. A nested PCR system with novel specific primers was designed based on the conserved regions of the SSU rRNA gene of *T. muris*. The nested PCR system for *T. muris* showed good specificity and high sensitivity for at least 100 *T. muris* trophozoites/mL and 0.1 ng/μL of fecal genomic DNA, which means that 176 trophozoites per gram of mouse feces could be detected. When using this nested PCR system, the detection rate was 18.96% (58/306), which was higher than the detection rate of 14.05% (43/306) detected via smear microscopy in fecal samples from five mouse strains. The sensitivity and specificity of nested PCR in detecting *T. muris* was found to be 100%, and it demonstrated a 26% increase in diagnostic sensitivity compared to the smear microscopy method in the present study. In conclusion, the nested PCR developed with novel primers based on the SSU rRNA gene of *T. muris* has good accuracy, specificity, and sensitivity for the detection of *T. muris* infections in laboratory mice.

## 1. Introduction

*Tritrichomonas muris* (*T. muris*), which belongs to the large group *Tritrichomonadea* of the phylum *Parabasalia*, is a unicellular, aerotolerant, flagellated protozoan parasite that was once considered a conditional pathogenic pathogen or a nonpathogenic member of murine flagellated protozoans in wild and laboratory mice [1,2,3,4]. However, studies have found that *T. muris* infections are related to host immune responses, which, nowadays, can change mucosal T-cell homeostasis and susceptibility to colitis [4]. As a result of *T. muris* infections, the tumor suppressor p53 can lead to an intestinal type 2 immune response [5]. Thus, *T. muris* infections seriously endanger the health of laboratory mice and test indicators.

*T. muris* mainly parasitizes the large intestines, especially the ceca, of different rodent species [1,6], including mice, rats, and hamsters [2,7,8,9,10]. *T. muris* is widespread worldwide, especially in Asia, North America, and Europe, where the infection rate is as high as 100 percent [8,9,10]. In addition, a mixed infection was observed in the intestinal tract of animals, which were simultaneously infected not only with *T. muris* but also with *G. duodenalis*, *P. hominis*, *Cryptosporidium* spp., and other parasites [11,12].

Currently, *Trichomonas* detection depends on fecal smear microscopic examinations and cultures, which have been regarded as the traditional detection method for many decades [13,14]. Although smear microscopy is capable of quick initial detection, its detection rate is low [15]. Researchers have reported that the 28 s rRNA gene of *T. muris* can be amplified via qPCR for species identification and classification [16]. The disadvantages of qPCR include the requirement for professional instruments, the use of fluorescent dyes, and the high demand for primers [17]. However, smear microscopy remains the main method of detection in clinical settings. Thus, to date, there are no on-site, reliable, and sensitive pathogen-detecting methods for *T. muris* infections in experimental animals.

The small subunit ribosomal RNA (SSU rRNA) gene, or the 16S-like ribosomal RNA gene, has proven to be an invaluable tool in molecular phylogenetic studies based on specific characteristics, such as its ubiquity, size, and high conservatism [18]. This molecule has accounted for numerous new insights in evolutionary biology, and its impact on our understanding of the origin and diversity of eukaryotes has been tremendous [19,20,21]. Due to its popularity as a molecular marker, SSU rRNA is widely used to detect and identify different genetic sequences of protozoans, such as *T. vaginalis*, *Tritrichomonas foetus* (*T. foetus*), *G. duodenalis*, and *Cryptosporidium* spp., but not *T. muris* [22,23,24,25,26,27,28]. Therefore, it is necessary to design a novel primer for clinical epidemiological studies of this pathogen based on SSU rRNA.

Nested PCR exhibits better performance with a high degree of sensitivity, specificity, repeatability, and reliability, and it eliminates the possibility of false positive signals [29]. Here, our study offers novel primers based on the SSU rRNA gene of *T. muris* to develop a nested PCR system that is specific and sensitive for the detection of *T. muris* infections in laboratory mice. This will make an important contribution to the field and ensure the quality of laboratory mice and experimental results.

## 2. Materials and Methods

### 2.1. Ethics Statement

All collection procedures were conducted in strict accordance with the guidelines of the Animal Care and Welfare Committee of Jilin University (IACUC Permit Number: 201606122).

### 2.2. Collection of Fecal Samples and Preparation of Genomic DNA

Randomly selected laboratory mice were provided by experimental animal breeding companies in different regions of China. Genomic DNA from fresh fecal samples was extracted using the QIAamp DNA stool mini kit (Qiagen, Valencia, CA, USA), according to the manufacturer’s instructions.

Additionally, other parasite genomic DNA, including *P. hominis*, *G. duodenalis*, *Cryptosporidium tyzzeri* (*C. tyzzeri*), *T. vaginalis*, *Escherichia coli* (*E. coli*), *Trichomonas gallinae* (*T. gallinae*) and *Salmonella typhimurium* (*S. typhimurium*), was extracted using the TIANamp Genomic DNA Kit (Tiangen, Beijing, China) and preserved at −40 °C in our laboratory.

### 2.3. Isolation of T. muris

The isolation and culture of *T. muris* were performed using a modified protocol described in the literature by other researchers [4,30,31]. Briefly, fecal contents were harvested from mice, passed through a 40 µm filter, and washed three times with PBS. The *T. muris*-containing mixtures were further treated through gradient centrifugation in Percoll (GE Healthcare, Uppsala, Sweden) at 1000× *g* for 15 min, and the purified *T. muris* trophozoites were collected from the interface using between 40% Percoll (GE Healthcare, Uppsala, Sweden) and 80% Percoll (GE Healthcare, Uppsala, Sweden). The number and viability of the isolated *T. muris* were determined by counting them using a hemocytometer.

*T. muris* was cultured under a density of 5 × 10^5^ trophozoites per mL of complete growth media, which consisted of Trichosel^TM^ broth (Becton Dickinson, San Jose, CA, USA) supplemented with 10% heat-inactivated horse serum (Gibco, Grand Island, NY, USA), 1% amphotericin B (Yuanye, Shanghai, China), 1% gentamicin (Yuanye, Shanghai, China), 1% penicillin (Yuanye, Shanghai, China), 1% streptomycin (Yuanye, Shanghai, China), and 1% vancomycin (Yuanye, Shanghai, China), and the pH was adjusted to 7.0. The *T. muris* trophozoites were cultured at 37 °C in a constant-temperature culture. The genomic DNA of *T. muris* trophozoites was extracted using a TIANamp Genomic DNA Kit (Tiangen, Beijing, China) and preserved at −40 °C.

### 2.4. Light Microscopy Observation

*T. muris* was observed microscopically using an Olympus CX43 microscope (Olympus, Tokyo, Japan) and photographed using an Olympus DP-71 camera (Olympus, Tokyo, Japan) with DP Controller 3.1.267 software. Pictures of *T. muris* at different staining morphologies were recorded at 400 or 1000 times magnification.

### 2.5. Scanning Electron Microscopy Observation

For SEM sample preparation, *T. muris* was cultured to 10^5^ per mL in Trichosel^TM^ broth (Becton Dickinson, San Jose, CA, USA) at 37 °C, collected via centrifugation at 1000× *g* for 10 min, and then, washed three times with PBS. A cell climbing test was conducted using a modified protocol described by other researchers’ literature [4,30,31]. The glass slides were pretreated with acetic acid for 2 h and absolute ethanol for 1 h, and washed with PBS 3 times. Post-drying, the slides were wrapped in aluminum foil and baked at 180 °C for 2 h in an oven (Thermo, Waltham, MA, USA). *Tritrichomonas muris* was isolated from the cecal contents of WT mice as described above, and suspended in PBS. 1.0 × 10^6^ *T. muris* trophozoites were seeded on poly-L-lysine-coated coverslips, cultured for 1 h at 37 °C in incubators (Thermo, Waltham, MA, USA), and fixed in 2.5% glutaraldehyde (Solarbio, Beijing, China) in a 0.1 M cacodylate buffer (Solarbio, Beijing, China), pH 7.2. Following 3 buffer rinses, the protozoa were filtered through polycarbonate membrane filters (1.2 μm, Millipore, Darmstadt, Germany), post-fixed for 30 min in 1% OsO4 (Aladdin, Shanghai, China) in 0.1 M cacodylate buffer (Solarbio, Beijing, China), and dehydrated in a graded series of ethanol. The dry filter pieces were attached to stubs and subsequently coated with gold. The morphologies of the anterior flagellum (AF), recurrent flagellum (RF), undulating membrane (UM), and axostyle (AX) in *T. muris* were examined under an S-3400N scanning electron microscope (SEM, S-3400N; Hitachi, Tokyo, Japan).

### 2.6. Primer Design

Nested PCR primers were designed using the Premier 5.0 software based on conserved region sequences of the SSU rRNA gene deposited in GenBank (accession No. AY886846.1). The first-round PCR primer sets were as follows: the forward primer F1: 5′-CTG TGA ACA AAT CAG GAC GCT-3′, and the reverse primer R1: 5′-ACC TTT GTG CGT ACA CTC CG-3′. The second-round PCR primer sets were as follows: the forward primer F2: 5′-GAT TCA GAT AAC GAG CGA GAT T-3′, and the reverse primer R2: 5′-CCT TTG TGC GTA CAC TCC G-3′. The primers were synthesized by Comate Biological Technology Co., Ltd. (Changchun, China).

### 2.7. Optimization of the Nested PCR Reaction System

The nested PCR reaction system was mainly optimized through the following two aspects: Mg^2+^ concentrations and annealing temperatures. The nested PCR assays were performed at Mg^2+^ concentrations of 0.5, 1.0, 1.5, 2.0, 2.5, 3.0, 3.5, 4.0, 4.5, and 5 mmol/L, respectively. The annealing temperatures of the first round were set with the following 10 gradients: 56, 57.0, 58.0, 59.0, 60.0, 61.0, 62.0, 63.0, 64.0, and 65.0 °C, respectively. The annealing temperatures of the second round were set with the following 10 gradients: 51.0, 52.0, 53.0, 54.0, 55.0, 56.0, 57.0, and 58.0 °C, respectively.

The reaction system was prepared as follows: The first-round PCR mixture (50 μL) contained 0.25 μL of Taq DNA polymerase (5 U/μL; Takara, Dalian, China), 5 μL of 10 × PCR buffer without Mg^2+^, 4 μL of dNTP mixture (2.5 mM each), 1 μL of forward and reverse primers (10 pmol/μL), 1 μL of a *T. muris* genomic DNA template, and different concentrations of MgCl_2_. In the second round of PCR, 1 µL of the PCR product from the first round was used as the DNA template for the PCR reaction, which included a standard PCR reaction mixture.

The reaction program was set as follows: pre-denaturation at 95 °C for 5 min, followed by 30 cycles of denaturation at 95 °C for 50 s, annealing at 55 °C for 50 s, extension at 72 °C for 1 min, and a final extension at 72 °C for 12 min. In order to avoid contamination, PCR was carried out under stringent conditions, as described previously [32,33]. Each batch of samples being analyzed contained both negative and positive controls. A standard PCR reaction, which contained water instead of sample, was included as a negative control, while the positive control contained 10 ng of *T. muris* genomic DNA instead of sample. The amplified products were electrophoresed on 1% agarose gel and stained with ethidium bromide to visualize the bands.

### 2.8. Sequence Analysis

The purified PCR product was cloned into the pMD-18T vector (TaKaRa, Dalian, China) and transformed into *E. coli* DH5α competent cells, to obtain the recombinant plasmid pMD-18T-muris. The single-cloning recombinant pMD-18T-muris bacterial colony was identified through PCR, and sequenced by Comate Biological Technology Co., Ltd.

The *T. muris* gene sequences were submitted to NCBI (Genbank No. OP437559), amplified via nested PCR, and cloned using *E. coli* DH5α. The SSU rRNA gene sequences were downloaded from NCBI, as shown in Table 1. Multiple sequence alignment was used to construct a phylogenetic tree using the maximum likelihood (ML) method. The phylogenetic tree was constructed using the MEGA software (version 6.0) [34].

### 2.9. Validation of the Nested PCR Assays

#### 2.9.1. Specificity Validation of the Nested PCR Reaction

In order to determine the specificity of the nested PCR reaction for *T. muris*, different parasites’ genomic DNA, including *P. hominis*, *G. duodenalis*, *C. tyzzeri*, *T. vaginalis*, *E. coli*, *T. gallinae*, and *S. typhimurium* was used for the nested PCR assays for *T. muris*, while the recombinant plasmid pMD-18T-muris was used as a positive control and ddH_2_O was set as a negative control. The reaction system and program were performed as described above.

#### 2.9.2. Sensitivity Validation of the Nested PCR Reaction

In order to evaluate the sensitivity of the nested PCR reaction for the detection of fecal mouse samples infected by *T. muris*, mouse feces were used to extract DNA. The feces were homogenized on a homogenizer (Huxi, Shanghai, China). The genomic DNA was extracted from the fecal samples using a QIAamp DNA stool mini kit (Qiagen, Valencia, CA, USA), according to the manufacturer’s instructions. The genomic DNA concentration of the mouse fecal samples infected by *T. muris* was measured, after which it was diluted as follows: 200 ng/μL, 100 ng/μL, 10 ng/μL, 1 ng/μL, 0.1 ng/μL, 0.01 ng/μL, 0.001 ng/μL, and 0.0001 ng/μL. The sensitivity of the nested PCR reactions was determined using the above genomic DNA with different concentrations as templates (1 μL for each reaction), while the recombinant plasmid pMD-18T-muris was used as a positive control. The PCR products were resolved by using electrophoresis on 1% agarose gel and stained with ethidium bromide. Subsequently, the number of genomic DNAs equivalent to the number of *T. muris* parasites per gram of feces was converted.

In addition, the detection sensitivity of *T. muris* trophozoites was evaluated using nested PCR reactions. The trophozoite cells were counted and diluted with sterilized ddH_2_O to final concentrations ranging from 10^6^ to 10^0^ trophozoites/mL with a ten-fold gradient dilution. Genomic DNA was extracted from 1 mL of the trophozoites and used as template DNA in the nested PCR assay, while the recombinant plasmid pMD-18T-muris was used as a positive control. The PCR products were resolved via electrophoresis on 1% agarose gel and stained with ethidium bromide to observe the sensitivity to different *T. muris* quantities.

### 2.10. Clinical Application of the New Primer in the Nested PCR System for T. muris

In order to evaluate the application effect of the new primers in the nested PCR of *T. muris*, 306 feces samples of laboratory mice from different experimental animal breeding companies in China were detected via smear microscopy and nested PCR. The feces samples were collected from March 2021 to July 2022, of which 60 were collected from C57/BL-6J mice, 60 from Kunming mice, 60 from BALB/C mice, 60 from Gerbil mice, and 66 from BALB/C nude mice, respectively. Firstly, the fresh feces of each laboratory mouse were collected and divided into two parts. One part of the feces was detected via smear microscopy (three smears were made for each sample), and the detection rate of *T. muris* was counted and analyzed. Another part of the feces was used for the fecal genomic DNA extraction and nested PCR detection of *T. muris*. Additionally, a statistical analysis was carried out on the infection rate of *T. muris.*

### 2.11. Data Analysis

A statistical analysis was performed using the SPSS software version 20.0 (IBM, Armonk, NY, USA). A chi-square test was used to estimate the statistical significance of the laboratory mice’s fecal samples collected from different species. All statistical tests were two-sided. Odds ratios (ORs) with 95% confidence intervals were used to assess the strength of associations. All ORs were adjusted for both age and sex. A value of *p* < 0.05 was considered statistically significant.

## 3. Results

### 3.1. Morphological Identification of T. muris

**Figure 1:**
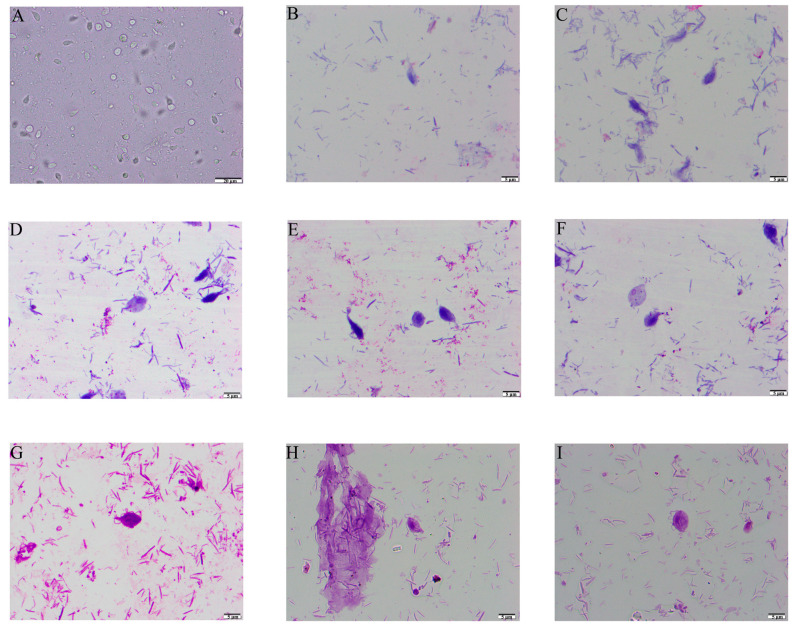
Light microscopy observation of *T. muris*. (**A**) Optical microscope observation with 400 times magnification. (**B**,**C**) Swiss staining observation with 1000 times magnification. (**D**–**H**) Swiss-Giemsa staining observation with 1000 times magnification and the nuclei were stained in blue. (**G**–**I**) Flagella-staining observation with the anterior flagellum and the posterior flagellum stained in blue, and the nuclei stained in dark blue.

**Figure 2:**
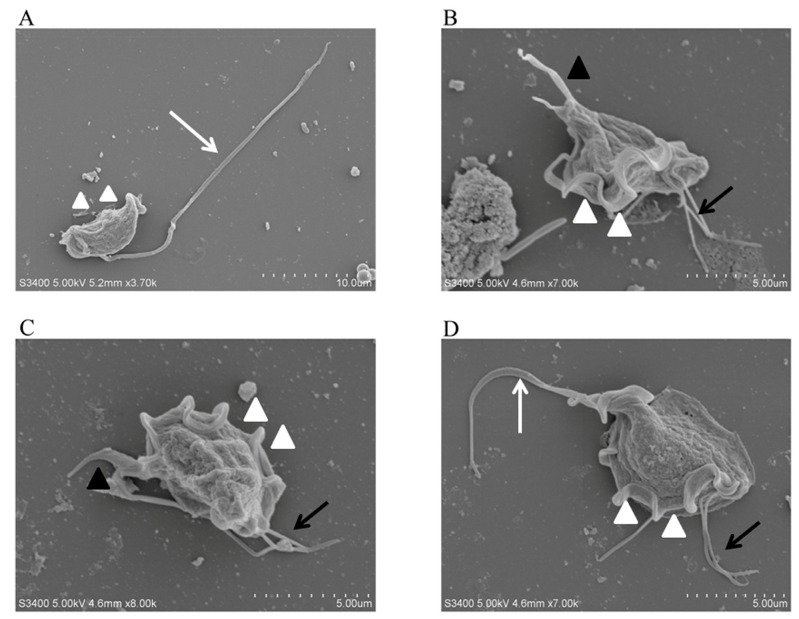
SEM observation of *T. muris*. The morphology of *T. muris* under different conditions was observed using a scanning electron microscope (SEM). (**A**–**D**) The white arrows represent the recurrent flagellum (RF), the white triangles represent the undulating membrane (UM), the black arrows represent the anterior flagella (AF), the black triangles represent the axostyle (AX).

*T. muris* was isolated and purified from the stools of laboratory mice and observed using smear microscopy. The results revealed that *T. muris* has a pear-shaped body and contains three anterior flagella, a recurrent flagellum, and nuclei structures (Figure 1). The SEM results showed that *T. muris* is a pear-shaped cell that is free-living and free-swimming. Three flagella protrude from the region of the pelta at the anterior end of the trophozoid cell. The recurrent flagellum, which is attached to the undulating membrane, runs along the entire cell surface toward the posterior end (Figure 2).

### 3.2. Sequence Analysis of T. muris’ Small Subunit Ribosomal RNA (SSU rRNA)

**Figure 3:**
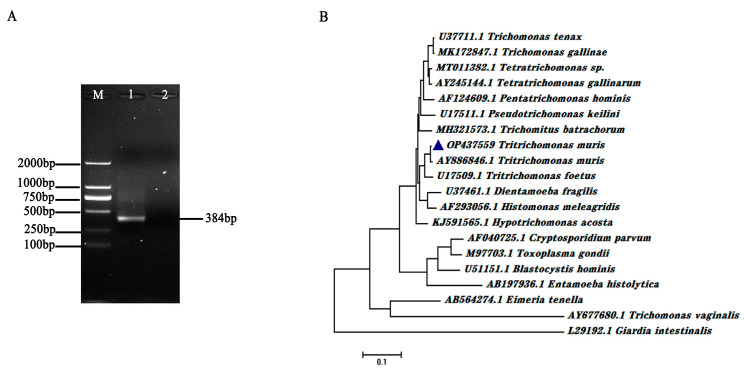
Amplification performance of the nested PCR primer sets and sequence alignment of *T. muris* SSU rRNA gene. (**A**) Validation of the SSU rRNA nested PCR primer sets using isolated *T. muris* genomic DNA as templates. The amplification products were detected through agarose gel electrophoretic analysis. M represents DL2000 DNA Marker. (**B**) Sequence alignment of isolated *T. muris* SSU rRNA and that in other parasites, including *Tririchomonas muris* (AY886846.1), *Tritrichomonas foetus* (U17509.1), *Trichomonas tenax* (U37711.1), *Pentatrichomonas hominis* (AF124609.1), *Dientamoeba fragilis* (U37461.1), *Entamoeba histolytica* (AB197936.1), *Cryptosporidium parvum* (AF040725.1), *Blastocystis hominis* (U51151.1), *Toxoplasma gondii* (M97703.1), *Eimeria tenella* (AB564274.1), *Giardia intestinalis* (L29192.1), *Trichomonas vaginalis* (AY677680.1), *Trichomonas gallinae* (MK172847.1), *Tetratrichomonas* sp. (MT011382.1), *Tetratrichomonas gallinarum* (AY245144.1), *Pseudotrichomonas keilini* (U17511.1), *Histomonas meleagridis* (AF293056.1), *Trichomitus batrachorum* (MH321573.1), and *Hypotrichomonas acosta* (KJ591565.1). The blue triangles represent the SSU rRNA gene sequence of the isolated *T. muris* (OP437559).

The SSU rRNA gene of *T. muris* was amplified using nested PCR assays and designed primers. The amplicon sizes of the first- and the second-round PCR products were 906 and 384 base pairs, respectively. A clear band with an expected size was obtained through agarose gel electrophoretic analysis (Figure 3A). The nested PCR-amplified products were subsequently cloned into pMD-18T and sequenced. The sequence alignment results revealed that the obtained SSU rRNA gene sequence isolated from the experimental mice belonged to the *Tritrichomonas* branch and shared a 98% gene sequence identity with the SSU rRNA gene of *T. muris* (Genbank No. AY886846.1; Figure 3B).

### 3.3. Determination of the Optimal Nested PCR

**Figure 4:**
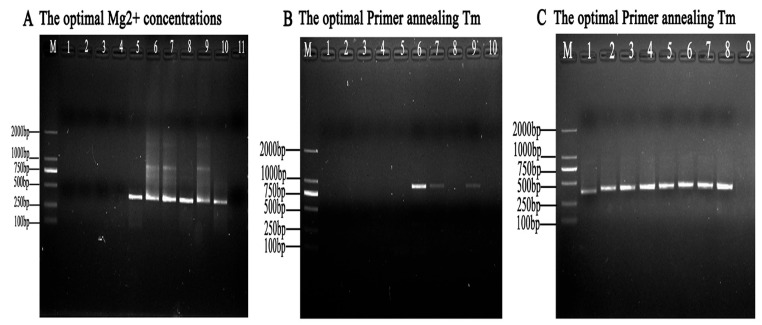
Optimization of the nested PCR system parameters. (**A**) Optimization of the Mg^2+^ concentrations. The nested PCR assays were carried out with Mg^2+^ concentrations of 0.5, 1.0, 1.5, 2.0, 2.5, 3.0, 3.5, 4.0, 4.5, and 5 mmol/L (lanes 1 to 10), respectively. (**B**) Optimization of the first-round annealing temperature in the nested PCR reaction system. The annealing temperature was set with 10 gradients. Lanes 1 to 10 indicate 56, 57.0, 58.0, 59.0, 60.0, 61.0, 62.0, 63.0, 64.0, and 65.0 °C, respectively. (**C**) Optimization of the second-round annealing temperature in the nested PCR reaction system. The annealing temperature was set with 10 gradients. Lanes 1 to 8 show 51.0, 52.0, 53.0, 54.0, 55.0, 56.0, 57.0, and 58.0, respectively. M represents DL2000 DNA Marker.

Mg^2+^ concentrations and annealing temperatures were the two key factors for improving the performance of Taq DNA polymerase amplification and primer–template combinations. The Mg^2+^ concentration optimization results showed that the nested PCR assays could obtain target bands when Mg^2+^ concentrations were in a range from 1.0 mmol/L to 5.0 mmol/L, and the brightness of the target bands first displayed an increased, and then, a decreased trend along with the Mg^2+^ concentration changes. The amounts of amplification products peaked when using 4.0 mmol/L Mg^2+^ (Figure 4A).

To justify the reliability of the nested PCR reaction system, different ranges of annealing temperature values were optimized. By comparing the Tm values of the primer, the following Tm ranges were established for each group of nested PCR reactions: For the determination of the optimal temperature screen, the first-round PCR results showed that a weak target band occurred at 61.0 °C (Figure 4B). The second-round PCR results showed that targeted bands were seen with annealing temperatures ranging from 51.0 °C to 58.0 °C (Figure 4C). Finally, the optimal annealing temperature was set to 56.0 °C (Table 2).

### 3.4. Specificity of the Nested PCR System

**Figure 5:**
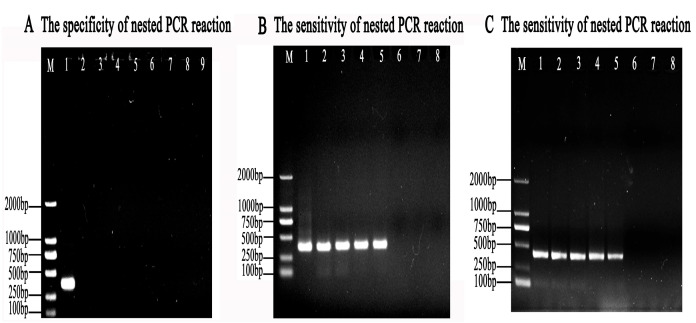
The specificity and sensitivity of the nested PCR system. (**A**) The specificity of the nested PCR system for *T. muris* was evaluated using pMD-18T-muris as a positive control template (lane 1); ddH_2_O as a negative control template (lane 2); and *G. duodenalis* (lane 3), *C. tyzzeri* (lane 4), *E. coli* (lane 5), *P. hominis* (lane 6), *T. vaginalis* (lane 7), *T. gallinae* (lane 8), and *S. typhimurium* (lane 9) as the tested templates. M represents the DL2000 DNA Marker. (**B**) The sensitivity of the second-round nested PCR for mice feces infected by *T. muris* was detected using template concentrations of 200, 100, 10, 1, 0.1, 0.01, 0.001, and 0.0001ng/μL genomic DNAs (lane 1–lane 7), respectively. (**C**) The sensitivity of the second-round nested PCR system for *T. muris* was measured using DNA of 10^6^, 10^5^, 10^4^, 10^3^, 10^2^, 10^1^, and 10^0^ *T. muris* (lane 1–lane 7), respectively. Lane 8 represents no template control. M represents the DL2000 DNA Marker.

To ensure the specificity of the nested PCR reaction, different species of parasites were selected and used for this assay. The results showed no target bands when using *P. hominis*, *G. duodenalis*, *C. tyzzeri*, *T. vaginalis*, *E. coli*, *T. gallinae*, and *S. typhimurium* as templates, while clear bands with a size of 384 bp occurred with the introduction of *T. muris*, which illustrated that the established nest PCR assays are highly specific to *T. muris* and had no cross-reactions with other parasites (Figure 5A).

### 3.5. Sensitivity of the Nested PCR

At least 0.1 ng/μL of genomic DNA of the mouse feces infected with *T. muris* could be detected using the nested PCR system (Figure 5B), which amounted to 176 trophozoites/g of feces.

In addition, the genomic DNA of at least 100 *T. muris* trophozoites/mL could be detected using the nested PCR system according to gel electrophoresis after PCR amplification (Figure 5C).

### 3.6. Clinical Application of the New Primer of the Nested PCR System for T. muris

The overall infection rate of *T. muris* measured using nested PCR was 18.96% (58/306), while the infection rate detected via smear microscopy was 14.05% (43/306), which was lower than that of the nested PCR. The infection rates of *T. muris* in C57/BL-6J mice detected using nested PCR were 13.33% (8/60) and 8.33% (5/60) via smear microscopy examination (χ^2^ = 6.708; df = 1; *p* < 0.01). The infection rates of *T. muris* in Kunming mice detected using nested PCR were 21.67% (13/60) and 20% (12/60) via smear microscopy examination (χ^2^ = 2.048; df = 1; *p* > 0.01). The infection rates of *T. muris* in BALB/C mice detected using nested PCR were 20% (12/60) and 13.33% (8/60) via smear microscopy examination (χ^2^ = 2.727; df = 1; *p* < 0.05). The infection rates of *T. muris* in Gerbil mice detected using nested PCR were 33.33% (20/60) and 26.67% (16/60) via smear microscopy examination. The infection rates of *T. muris* in BALB/C nude mice detected using nested PCR were 7.5% (5/60) and 3.03% (2/60) via smear microscopy examination (χ^2^ = 13.11; df = 1; *p* < 0.01) (Table 3).

The present study determined the sensitivity and specificity of nested PCR in detecting *T. muris* to be 100%, with no instances of false negative or false positive samples. In contrast, smear microscopy exhibited a sensitivity percentage of 74% and a specificity percentage of 100% in the clinical diagnosis of *T. muris*, with 15 false negative samples and no false positive samples. The nested PCR method demonstrated a 26% increase in diagnostic sensitivity compared to the smear microscopy method in the clinical application of detecting *T. muris* in 306 laboratory mice (Table 4).

## 4. Discussion

The majority of *Trichomonas* monitoring was based on smear microscopy. In the case of poor experimental conditions, the sensitivity of direct smear detection averages between 50% and 60% [35,36,37]. Smear microscopy is fast and inexpensive but has low sensitivity and specificity [38]. For the sake of abandoning the shortcomings of smear microscopy, the detection of *Trichomonas* using nested PCR was developed, which has the advantages of accurate reaction, excellent specificity, and high sensitivity [39,40,41,42]. In this study, two rounds of nested PCR were established to screen for *T. muris* infections in the digestive tracts of mice. Through sensitivity detection, the genomic DNA of at least 100 *T. muris* trophozoites/mL and 0.1 ng of genome DNA of mouse feces infected by *T. muris* per μL, which amounted to 176 trophozoites/g of feces, could be detected. Additionally, there were no cross-reactions with *P. hominis*, *G. duodenalis*, *C. tyzzeri*, *T. vaginalis*, or *T. gallinae*. The nested PCR improved the sensitivity of detecting *T. muris* by 26% over smear microscopy examination, and its sensitivity was significantly higher than that of smear microscopy examination. It is important for clinical applications to detect *T. muris* infection rates and prevalence. In addition, clinical studies have shown that using nested PCR to detect *Trichomonas* in bronchoalveolar lavage fluid significantly increases its sensitivity percentage compared to smear microscopy examination [43]. Moreover, *Trichomonas vaginalis* diagnosis was based on wet mount microscopy, which has poor sensitivity (60%) compared with PCR assays [44]. The results of this study indicated that nested PCR reactions have higher sensitivity and specificity. As a result of using the PCR method, Da Costa et al. [45] were able to detect the ITS gene of *T. muris*, and the PCR product length was 65 bp, which was too short to be confused with a dimer or primer. For this PCR method, high electrophoresis time and operation requirements were needed; otherwise, the electrophoresis gel would have easily escaped. However, the nested PCR product in this study was 384 bp, which was moderately sized and could avoid this problem. Additionally, nested PCR was more sensitive for detecting *Trichomonas* genes in clinical applications than PCR [46,47]. But Escalante NK et al. [4] used the qPCR method to detect 28 s rRNA genes of *T. muris***,** which has the disadvantages of high cost and high instrument requirements, and many laboratories cannot obtain relatively expensive qPCR machines. One key disadvantage of existing qPCR techniques is their reliance on the reference standard curve, which makes comparing qPCR findings between labs challenging [48,49]. However, the nested PCR method used in this study to detect *T. muris* avoids the above issues in clinical application.

It is known that PCR is a tool of high sensitivity and specificity, particularly for identifying morphologically indistinguishable parasites such as species of *Giardia* and assemblages of *Cryptosporidium*, and for detecting their genetic variation [50]. Extensive genetic variation was observed within *Giardia*, of which there have been at least eight assemblages (A-H) identified in *G. duodenalis*. As a result of the genetic diversity of the *Giardia* population, this allows us to investigate *Giardia duodenalis*’ potential ecologically and evolutionarily [51]. Similarly, there is extensive genetic variation within the genus *Cryptosporidium*, with more than 120 genotypes of *Cryptosporidium* detected by analyzing the SSU rRNA gene [52]. Additionally, nested PCR is a considerably more specific and sensitive technique than PCR, so nested PCR was used to investigate the genetic variability within *T. muris* [50]. Based on the results of this study, one genotype of *T. muris* was identified, which indicates that it has a relatively wide distribution and stable transmission pattern. It will be necessary to conduct further research and exploration on other genotypes and the genetic variability of *T. muris*.

The commonly used homologous evolutionary analysis of conservative genes in intestinal protozoa mainly includes the 18S rRNA and ITS gene sequences. For example, the 18S gene and ITS gene were used to analyze the epidemic situation and homologous evolution of *P. hominis* [53,54]. SSU rRNA, as a conserved gene, has been widely used in phylogenetic comparisons; e.g., the SSU rRNA gene was used to perform a phylogenetic analysis among the genera of *Trichomonas* by comparing the base sequence length and secondary structure characteristics in *T. foetus* and *T. vaginalis* [22,23,24,55]. In addition, it was used to analyze the genetic characterization of *Tritrichomonas gallinae* (*T. gallinae*), *Tritrichomonas tenax* (*T. tenax*), and *Tritrichomonas canistomae* (*T. canistomae*), which were isolated from European turtle doves (*Streptopelia turtur*) and racing pigeons (*Columba livia*) [56]. PCR amplification and sequence analysis using SSU rRNA as a reference gene were helpful for investigating its prevalence in herbivores (cattle, sheep, and horses) in different regions of the world [57,58]. There are at least 44 effective species of *Cryptosporidium* and more than 120 genotypes of *Cryptosporidium* in humans and animals that have been detected by analyzing the SSU rRNA gene [52]. Furthermore, in this study, a novel primer for the detection of *T. muris* infections using nested PCR based on SSU rRNA was designed, which exhibited higher specificity and sensitivity when compared to the classical method of smear microscopy.

Cross-infection of intestinal parasites occurs in experimental mice, and, sometimes, it is difficult to distinguish between them using common smear microscopy. The most dramatic illustration of this is the phenomenon of a mixed infection of intestinal protozoa that exists in the intestinal tracts of laboratory mice. Laboratory mice can be infected with *G. duodenalis* and *Cryptosporidium*, which can parasitize their intestinal tracts [59,60]. *T. muris* and *P. hominis* are two kinds of flagellate parasites that can parasitize the ceca of rats, mice, and hamsters and have similar morphological characteristics. According to reports by Escalante et al. (2016) and Ghindilis et al. (2019) [4,17], *T. muris* showed morphological characteristics similar to those described in this study. It appears pear-shaped with an anterior vesicular nucleus. Three anterior flagella arise from the anterior region, as well as a posterior flagellum that is attached to its body by means of an undulating membrane that continues posteriorly as a free flagellum. It has a large oval-shaped nucleus, a slit-like cytostome, and a sausage-shaped parabasal body in the anterior region. *P. hominis* is piriform, measuring 8 to 20 by 3 to 14 μm, and ordinarily has five anterior flagella plus one posterior flagellum [53,54]. However, the nested PCR-specific primers designed in this study are particularly effective in distinguishing *T. muris* from other trichomonas due to their specificity.

In fact, it is still a worldwide problem that experimental mice’s digestive tracts are infested with *T. muris*, which is a serious threat to their health. For example, according to a study by Won et al. (2006) [8], laboratory mice in Korea had a 69% prevalence of *T. muris* infections. Surprisingly, the infection rate of *Tritrichomonas* sp. was up to 100% in different animal houses in Brazil [10]. Similarly, Japanese mice were found to be exclusively infected with *T. muris*, with a prevalence of infection as high as 67.8% in some areas [9]. A number of previous studies have shown that *T. muris* infections are largely caused by fecal contamination and pseudocyst transmission [1,2]. In view of these findings, it may be beneficial to prioritize the need to strengthen the management of experimental animal housing and detect animal health whenever possible [61]. Therefore, to prevent *T. muris* infections, animal breeding laws and regulations should be improved [62,63,64]. The following aspects should be improved: (i) Increasing awareness of the importance of clean and SPF animal production and the health and epidemic prevention of animal houses [62,65,66]. (ii) Mouse breeding environments should be clean and dust-free with fresh air, temperatures of 18–22 °C, relative humidities of 50–60%, and noise below 85 decibels [67]. In addition to cleaning and disinfecting rat houses, mouse boxes, water dispensers, bedding, and food, regular pest control is required [68]. (iii) Regular parasite detection should be performed in laboratory mice. (iv) A regular monitoring schedule should be maintained for intestinal parasites, such as *P. hominis*, *G. duodenalis*, *Cryptosporidium* sp., and *T. muris*.

In the present study, novel and specific SSU rRNA primer sets were designed for *T. muris* detection in laboratory mice. These results clearly demonstrate that nested PCR has higher sensitivity, reliability, and specificity. Thus, the present study provides further understanding of novel approaches for the detection of *T. muris* and a new means to ensure experimental animal health and welfare.

## 5. Conclusions

In conclusion, a nested PCR system with new primers has successfully been developed, which has higher specificity and sensitivity for the detection of *T. muris* infections in laboratory mice. Therefore, these findings should make an important contribution to the field and ensure the quality of laboratory mice.

## Figures and Tables

**Table 1:** Information about species in homologous evolution.

Species Names	GenBank No.	Product Size (bp)	Taxonomy ID
*Tririchomonas muris*	AY886846.1	1502	5726
*Tritrichomonas foetus*	U17509.1	1569	1,144,522
*Trichomonas tenax*	U37711.1	1580	43,075
*Pentatrichomonas hominis*	AF124609.1	1504	5728
*Dientamoeba fragilis*	U37461.1	1676	43,352
*Entamoeba histolytica*	AB197936.1	1941	5759
*Cryptosporidium parvum*	AF040725.1	7820	5807
*Blastocystis hominis*	U51151.1	1770	12,968
*Toxoplasma gondii*	M97703.1	1795	5811
*Eimeria tenella*	AB564274.1	1829	5802
*Giardia intestinalis*	L29192.1	1844	5741
*Trichomonas vaginalis*	AY677680.1	2032	5722
*Trichomonas gallinae*	MK172847.1	1924	56,777
*Tetratrichomonas* sp.	MT011382.1	1624	1,851,209
*Tetratrichomonas gallinarum*	AY245144.1	1512	5730
*Pseudotrichomonas keilini*	U17511.1	1569	39,065
*Histomonas meleagridis*	AF293056.1	1602	135,588
*Trichomitus batrachorum*	MH321573.1	1496	5732
*Hypotrichomonas acosta*	KJ591565.1	1515	5735

**Table 2:** Optimal conditions of nested PCR reaction system.

Reaction System	Size of Amplified Fragment	Tm	Mg^2+^	No. of Cycles
The 1st PCR	906 bp	61.0 °C	4.0 mmol/L	30 cycles
The 2nd PCR	384 bp	56.0 °C	4.0 mmol/L	30 cycles

**Table 3:** Occurrence of *T. muris* infections in laboratory mice.

Category	No.	No. Positive (%)	No. Positive Using Microscope (%)	*p*-Value	df	χ^2^	% (95% CI)
C57/BL-6J mice	60	8 (13.33%)	5 (8.33%)	0.01	1	6.708	3.25 (1.295–8.136)
Kunming mice	60	13 (21.67%)	12 (20%)	0.152	1	2.048	1.808 (0.8–4.087)
BALB/C mice	60	12 (20%)	8 (13.33%)	0.099	1	2.7	2.0 (0.872–4.585)
Gerbil mice	60	20 (33.33%)	16 (26.67%)	-	1	-	Reference
BALB/C nude mice	66	5 (7.5%)	2 (3.03%)	0	1	13.1	6.1 (2.118–17.572)
Total	306	58 (18.96%)	43 (14.05%)				

**Table 4:** Diagnostic sensitivity and specificity of the assays used in this study for the diagnosis of *T. muris*.

	Microscope	Nested PCR
True positive samples	43	58
False negative samples	15	0
True negative samples	248	248
False positive samples	0	0
Diagnostic sensitivity	74%	100%
Diagnostic specificity	100%	100%

## Data Availability

No data were used for the research described in this article.
